# Stress Responses from the Endoplasmic Reticulum in Cancer

**DOI:** 10.3389/fonc.2015.00093

**Published:** 2015-04-20

**Authors:** Hironori Kato, Hideki Nishitoh

**Affiliations:** ^1^Laboratory of Biochemistry and Molecular Biology, Department of Medical Sciences, University of Miyazaki, Miyazaki, Japan

**Keywords:** endoplasmic reticulum, mitochondria-associated ER membrane, unfolded protein response, ER stress, cancer

## Abstract

The endoplasmic reticulum (ER) is a dynamic organelle that is essential for multiple cellular functions. During cellular stress conditions, including nutrient deprivation and dysregulation of protein synthesis, unfolded/misfolded proteins accumulate in the ER lumen, resulting in activation of the unfolded protein response (UPR). The UPR also contributes to the regulation of various intracellular signaling pathways such as calcium signaling and lipid signaling. More recently, the mitochondria-associated ER membrane (MAM), which is a site of close contact between the ER and mitochondria, has been shown to function as a platform for various intracellular stress responses including apoptotic signaling, inflammatory signaling, the autophagic response, and the UPR. Interestingly, in cancer, these signaling pathways from the ER are often dysregulated, contributing to cancer cell metabolism. Thus, the signaling pathway from the ER may be a novel therapeutic target for various cancers. In this review, we discuss recent research on the roles of stress responses from the ER, including the MAM.

## Introduction

**Figure 1:**
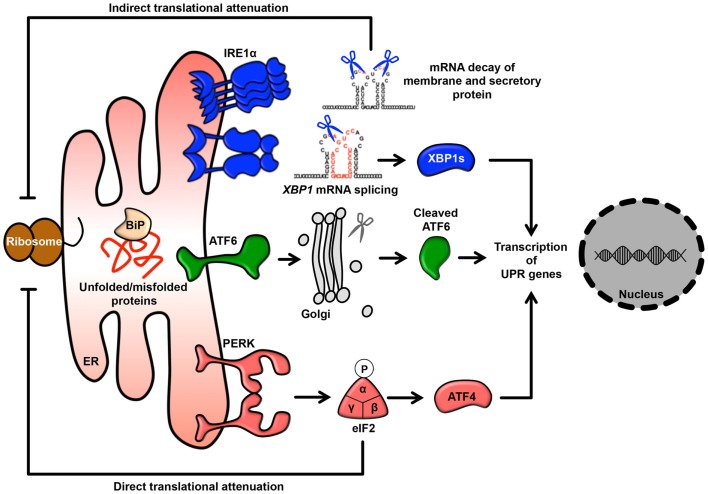
**The UPR signaling pathway**. Membrane and secretory proteins are synthesized by the rER and translocated into the ER lumen. Activated PERK phosphorylates eIF2α and causes general translational attenuation. The PERK-eIF2α pathway selectively induces expression of the transcription factor ATF4. Activated IRE1α undergoes dimerization and oligomerization, splices an intron from the XBP1 mRNA and produces the transcription factor XBP1s. IRE1α can also induce mRNA degradation. Activated ATF6 translocates to the Golgi and is sequentially cleaved by the Golgi-resident site-1 and site-2 proteases, thereby releasing cleaved ATF6.

In eukaryotic cells, the endoplasmic reticulum (ER) is an organelle that extends throughout the cytoplasm as a vast membranous network. The shape of the ER reflects its many cellular functions, which include folding newly synthesized proteins, calcium homeostasis, and phospholipid synthesis, leading to the regulation of various intracellular signaling pathways (1–6). In particular, as a central platform of protein quality control, the ER contributes to adaptation to adverse synthetic, metabolic, and other conditions. When the integrity of the ER is perturbed by adverse conditions, unfolded/misfolded proteins accumulate in the ER lumen, a condition called ER stress, which in turn activates the unfolded protein response (UPR) (7, 8). Furthermore, some reports have suggested that perturbation of calcium homeostasis and dysregulation of lipid metabolism are also involved in activation of the UPR (9–14). UPR signaling consists of three major stress sensors that reside on the ER membrane: RNA-dependent protein kinase-like kinase (PERK), activating transcription factor 6 (ATF6), and inositol-requiring enzyme 1α (IRE1α). The roles of these signaling pathways are to adapt to ER stress through translational attenuation, upregulation of ER chaperones, and protein degradation (8, 15) (Figure 1). Interestingly, activation of the UPR in cancer cells is sustained by exposure to various stresses, including hypoxia, oxidative stress, and nutrient starvation. Therefore, it is currently believed that the UPR may play critical roles in tumor progression, metastasis, tumorigenesis, and survival (16–18).

**Figure 2:**
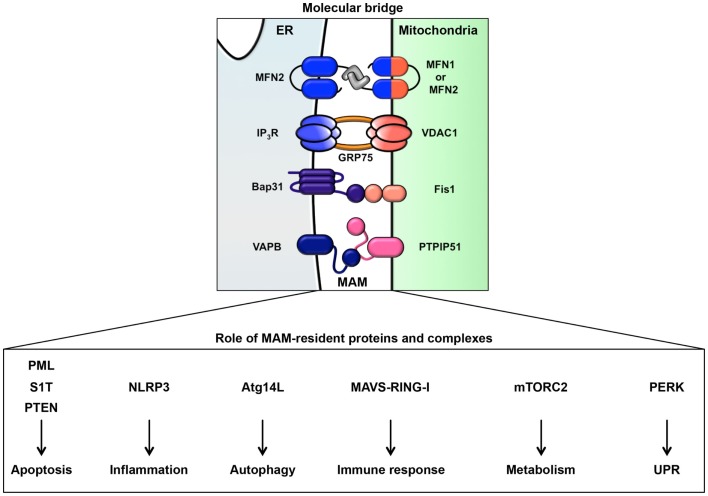
**ER-mitochondrion tethering**. In mammalian cells, four types of molecular bridges for ER-mitochondrion tethering have been identified. ER-resident MFN2 interacts with mitochondrial MFN1 and MFN2. The ER calcium channel IP_3_R associates with the mitochondrial calcium channel VDAC1 through GRP75. The ER protein Bap31 connects with the mitochondrial protein Fis1. The ER protein VAPB interacts with the mitochondrial protein PTPIP51. Indicated MAM-resident proteins and complexes regulate various signaling pathway from MAM as described in figure.

It has been observed that approximately 5–20% of the mitochondrial surface directly contacts the ER (19, 20). This site of close contact between the ER and mitochondria is called the mitochondria-associated ER membrane (MAM) and is formed by several molecular bridges. The mitochondrial outer membrane proteins mitofusin 1 and 2 (MFN1 and MFN2) are required for mitochondrial fusion. MFN1 contributes to mitochondrial docking and fusion, whereas MFN2 stabilizes associations between mitochondria. This maintenance of mitochondrial morphology is regulated by hetero-oligomeric MFN complexes on the mitochondrial outer membrane. Interestingly, MFN2 is also located on the ER membrane and forms homotypic or heterotypic complexes with mitochondrial MFNs, resulting in an interaction between the ER and mitochondria (21) (Figure 2). Acting as a similar molecular bridge, the inositol triphosphate receptor (IP_3_R) on the ER indirectly interacts with the mitochondrial outer membrane-resident voltage-dependent anion channel 1 (VDAC1) via the cytosolic chaperone glucose-regulated protein 75 (GRP75) (22). In addition, recent reports have demonstrated that the B-cell receptor-associated protein 31 (Bap31)-mitochondrial Fission-1 homolog (Fis1) complex (23) and vesicle-associated membrane protein-associated protein B (VAPB)-protein-tyrosine phosphatase interacting protein 51 (PTPIP51) complex act as tethering complexes for the ER-mitochondrion bridge (24) (Figure 2).

The MAM is crucial for the regulation of numerous cellular functions including lipid trafficking (25–27), calcium cycling (28, 29), and energy metabolism (30). This subdomain of the ER also directly contributes to biogenesis through the synthesis of phospholipids and sphingolipids, the levels of which are enriched at the MAM (25, 31–35). Interestingly, most cancer cells show characteristic alterations in *de novo* lipid biosynthesis, lipogenic phenotype, and lipid metabolism (36, 37). Therefore, current reports have considered that lipogenesis- and lipolysis-related pathways are involved in tumorigenesis, migration, invasion, and survival (36–38). The MAM has also been shown to act as a platform for various intracellular signaling pathways, including oncogenic signaling (39–41). Although several groups have addressed the pathophysiological relevance between the MAM and diseases, the role of the MAM, especially in cancer, has not been clearly elucidated. In this review, we will summarize some emerging roles of stress responses from the ER, particularly from the MAM, and discuss whether the MAM might be a potential therapeutic target in cancer.

## Signaling Pathways from the ER

Endoplasmic reticulum stress activates the UPR, which is the most thoroughly characterized stress signaling pathway from the ER. The UPR also plays important roles in tumor development and tumor growth through adaptation to microenvironments. In cancer cells, the UPR is constitutively activated because abnormal cell proliferation requires elevated protein synthesis during tumor development and tumor growth (42, 43). In addition, cancer cells are exposed to various stresses such as hypoxia, low glucose, low pH, and nutrient starvation, which are well known to induce the UPR (42–45). Recent research in oncology indicates that these microenvironmental challenges are associated with the constitutive activation of the UPR. Additionally, the perturbation of calcium homeostasis in cancer cells is correlated with their abnormal phenotypes, including sustained cell proliferation and avoidance of cell death, through the remodeling of calcium signaling (46–48). Furthermore, cancer cells undergo changes in lipid metabolism via the dysregulation of lipogenic and lipolytic enzymes, leading to lipid stress signaling. This is closely correlated with tumorigenesis, malignancy, and growth (36–38). Abnormalities in calcium homeostasis and lipid metabolism directly trigger ER stress (9–14). This section focuses on the cancer-related roles of the ER stress signaling pathways, including UPR signaling, calcium signaling, and lipid signaling.

## The UPR

Unfolded protein response signaling is mediated by three major transmembrane transducers: PERK, ATF6, and IRE1α (Figure 1). These sensors are maintained in an inactive state by binding to an ER chaperone, the 78 kDa glucose-regulated protein (GRP78), which is known as a master regulator of the UPR. During ER stress, GRP78 dissociates from sensor proteins in response to the accumulation of unfolded/misfolded proteins and reduced ER calcium content, resulting in the activation of distinct UPR signaling branches (8, 13, 49).

### The PERK branch

Protein kinase-like kinase is an ER type I transmembrane kinase that has a PEK-like catalytic domain in its cytosolic C-terminal region. The activated PERK pathway phosphorylates eukaryotic translation initiation factor 2α (eIF2α) and leads to the inhibition of protein translation into the ER (8, 15, 50). In addition, phosphorylation of eIF2α selectively induces the expression of activating transcription factor 4 (ATF4), thereby inducing growth arrest and upregulating UPR genes including CCAAT/enhancer-binding protein-homologous protein (CHOP), ER oxidoreductase 1α, and several pro-apoptotic factors (8, 15). Phosphorylation of eIF2α is also induced by multiple kinases, including protein kinase R, general control non-repressed 2, and haem-regulated eIF2α kinase. eIF2α phosphorylation-related signaling is described as the integrated stress response (ISR) (43, 51–53). The ISR is activated in response to various stresses, including amino acid starvation, viral infection, and haem deficiencies, in addition to ER stress. PERK also directly phosphorylates transcription factor NF-E2-related factor 2 (Nrf2), which is known to have the dual role in cancer, tumor suppressor function and oncogenic function (54). Under unstressed condition, Nrf2 maintains the inactive state in the cytoplasm through interaction with kelch-like ECH-associated protein 1. Phosphorylated Nrf2 by PERK dissociates from kelch-like ECH-associated protein 1, resulting in translocation into the nucleus and expression of antioxidant genes (55, 56). However, artificial activation of PERK mainly induces ISR signaling target genes in an eIF2α phosphorylation-dependent manner (57). Although PERK is activated by not only ER stress but also other stress (e.g., glucose starvation and oxidative stress), PERK-mediated Nrf2 activation may be limited to the ER stress condition.

Most cancers are constitutively exposed to various stresses, including hypoxia and nutrient deprivation, even when glycolysis and angiogenesis are promoted in cancer cells (42–45). Activation of PERK signaling and the ISR are considered necessary for tumor survival during hypoxia and nutrient starvation (43). Hypoxia and oxidative stress increase the generation of reactive oxygen species (ROS). In addition, ER oxidoreductase 1α, which regulates ER redox status, is upregulated through PERK signaling in the ISR (58–60). Consistent with the enhanced production of ROS in tumors, the expression of ER oxidoreductase 1α is substantially increased in various types of cancers (61). The ablation of PERK signaling or the ISR leads to ROS production and thereby impairs tumor growth through oxidative DNA damage (62). Furthermore, the ISR, which induces ATF4 expression, is also required for tumor survival and growth. Suppression of ATF4 expression inhibits tumor survival and proliferation in response to both amino acid deprivation and glucose deprivation (63, 64). Taken together, cancer cells adapt to hypoxia, oxidative stress, and nutrient starvation by improving their PERK- and ISR-mediated redox homeostasis and metabolic homeostasis, respectively. Transcription factor CHOP, a downstream target of ATF4, induces cell death in response to the intense and/or prolonged ER stress. Deletion of CHOP or suppression DNA damage-inducible 34, a downstream of CHOP, promotes tumor dedifferentiation and survival. Thus, it is possible that this dual role of the PERK and ISR signaling pathways might be therapeutic targets for cancer. Indeed, it has been reported that PERK specific inhibitor GSK2656157 impairs angiogenesis and amino acid metabolism, resulting in prevention of tumorigenesis *in vivo* (65). Furthermore, in leukemic cells, salubrinal, a selective inhibitor of eIF2α dephosphorylation induces synergistic apoptosis under proteasome inhibitor-induced ER stress condition (66). These observations strongly suggest that the compounds, which regulate PERK pathway, may shed light on the treatment of cancer.

### The IRE1α branch

Inositol-requiring enzyme 1α is an ER type I transmembrane endoribonuclease/kinase protein that contains a kinase domain and an endoribonuclease domain in its cytosolic C-terminal region. During ER stress, activated IRE1α catalyzes the splicing of a 26-nt intron from the mRNA of X-box binding protein 1 (XBP1), resulting in the production of active XBP1 (XBP1s) transcription factor and the consequent promotion of ER-associated degradation (8, 15). IRE1α also cleaves non-specific mRNAs through a process termed regulated IRE1α-dependent mRNA decay (RIDD) (8, 15, 67, 68). Several reports have indicated that the IRE1α signaling, especially via the IRE1α-XBP1 pathway, is required for tumor growth and survival during hypoxia. For example, XBP1-deficient cells are vulnerable to hypoxia-induced apoptosis, leading to the impairment of hypoxic growth in tumor xenografts (18). A recent report suggests that expression of the spliced form of XBP1s is essential for tumor angiogenesis independently of vascular endothelial growth factor, a well-known angiogenic factor (69). XBP1s is constitutively activated through the hypoxia-inducible factor-1α pathway in response to hypoxia, resulting in tumor development (70). In addition, a recent analysis indicated that the IRE1α-RIDD pathway contributes to tumor growth, infiltration, and invasion under tumor microenvironment conditions (71).

Inositol-requiring enzyme 1α also induces activation of c-Jun N-terminal kinase (JNK) through interaction with tumor necrosis associated factor 2 and apoptosis signaling-regulating kinase 1 (72, 73). IRE1α-JNK pathway contributes to not only apoptotic cell death (72) but also cell survival via c-Jun activation (74, 75), suggesting that IRE1α-JNK pathway has binary effects on cell fate. In neuroblastoma, IRE1α-JNK pathway induced by severe ER stress triggers apoptosis (76). On the other hand, IRE1α-JNK pathway involves in induction of autophagy, which protect cancer cell death (77). Thus, it is considered that IRE1α signaling contributes to both tumor survival and cell death in some situations of chronic stress. Since activities of kinase and endoribonuclease of IRE1α can be selectively controlled by distinct classes of adenosine triphosphate (ATP)-competitive inhibitors (78), it is expected that regulation of pathway might be a therapeutic strategy for cancer.

Endoplasmic reticulum stress activates T cells in the adaptive immune system in tumor (79). IRE1α signaling phosphorylates IκB kinase and activates NF-κB pathway, leading to induction of inflammatory response (80). Pro-inflammatory cytokines interleukin (IL)-6 and tumor necrosis factor-α are induced by XBP1s in response to ER stress (81, 82). In macrophages, activation of UPR is triggered by culture media conditioned by ER stressor-treated cancer cells via toll-like receptor, suggesting that cancer cells non-cell-autonomously induce tumor inflammation through the transmission of UPR signaling to macrophages (83). Recent study has shown that loss of ER chaperone calreticulin results in the prevention of phagocytosis and antigen presentation in dendritic cells, and translocation of calreticulin to the plasma membrane induced by anti-neoplastic drugs anthracycline triggers tumor immunogenicity (84). Although anti-tumor agent cisplatin has no effect on induction of an anti-cancer immune response via the UPR activation, the combination of ER stress inducer (e.g., thapsigargin or tunicamycin) with cisplatin triggers translocation of calreticulin to the plasma membrane and trigger tumor immunogenicity (85). Thus, it is possibility that combination of anti-tumor drugs and ER stress inducers might become therapeutic strategies for various cancers.

### The ATF6 branch

Activating transcription factor 6 is an ER type II transmembrane protein that possesses a transcriptional activation domain in its cytosolic region. Upon ER stress, ATF6 is transported from the ER membrane to the Golgi, where it is sequentially cleaved by the Golgi-resident site-1 and site-2 proteases. The cleaved ATF6 is translocated into the nucleus and binds to ER stress elements and UPR elements, leading to the expression of UPR target genes such as ER chaperones (8, 15). Expression of GRP78, which is upregulated by the ATF6 signaling during ER stress, has been shown to be essential for tumor growth, survival, progression, and metastasis (86–88). Conversely, overexpression of GRP78 suppresses non-steroidal anti-inflammatory drug-induced apoptosis in cancer cells (86). Therefore, both ATF6 signaling and GRP78 appear to contribute to tumor adaptation to microenvironmental challenges by promoting protein folding.

## Calcium Signaling from the ER

The ER is a major intracellular calcium store. Calcium contributes to a wide variety of intracellular signaling pathways as a second messenger (48, 89). However, depletion of calcium in the ER disturbs the function of ER chaperones and induces ER stress, leading to activation of the UPR (11, 13, 14). In addition, increasing intracellular calcium concentrations triggers cytotoxicity under certain conditions such as increased ROS generation (48). Thus, both cytosolic calcium and the concentration of calcium in the ER must be tightly regulated. Import of calcium into the ER is maintained by sarcoplasmic/ER calcium ATPase (SERCA) pumps, which function against a calcium concentration gradient (90). Conversely, ER-resident calcium is released through two tetrameric calcium channels, ryanodine receptors and inositol 1,4,5-trisphosphate receptors (IP_3_Rs) (91–93). These mediators are involved in protein synthesis, gene expression, secretion, the cell cycle, cell proliferation, and differentiation through the regulation of calcium signaling (48, 89, 94). Surprisingly, in most cancers, the calcium concentration in the ER is decreased, whereas the cytosolic calcium concentration is increased (47, 48). This indicates that various cancers alter the activity or expression of SERCA pumps and IP_3_Rs, resulting in the remodeling of calcium signaling. Because abnormal intracellular calcium homeostasis occurs via altered activity or expression of ER-resident calcium pumps and channels in cancer, regulation of the ER-resident calcium status may be a candidate for the treatment of cancer.

### Role of SERCA pumps in the remodeling of calcium signaling

Sarcoplasmic/ER calcium ATPase pumps, which are type P ATPase pumps, import calcium into the ER from the cytosol to regulate the cytosolic calcium level. They have diverged into three different genes and are further composed of at least five isoforms (SERCA1a, 1b, 2a, 2b, and 3). Among the SERCA pumps, SERCA2b is ubiquitously expressed and has the highest calcium affinity (29, 90, 95–97). Phospholamban and sarcolipin have been reported to be endogenous negative regulators that suppress the affinity of SERCA pumps for calcium (98). Furthermore, the activity of SERCA pumps is also regulated by post-translational modifications including SUMOylation, glutathiolation, and nitration (99–101). Several reports have shown that the expression of SERCA pumps, especially SERCA2b and SERCA3, is frequently decreased in cancer cells. Korosec et al. and Endo et al. reported that in colon, lung, and oral cancer, SERCA2b expression was reduced by alterations of the promoter region or by an epigenetic mechanism as an early event in tumorigenesis (102, 103). Although the mechanism remains unclear, the ATPase activity of SERCA2b is also reduced in advanced tumorigenic thyroid cells, consistent with the reductions at the mRNA and protein levels (104). Furthermore, germline alterations in SERCA2b have been indicated as closely involved in the early phase of carcinogenesis in the colon and lung (102). Similar to SERCA2b, SERCA3 is also reduced in colon and gastric cancer cells (105, 106). In addition, SERCA3 expression is involved in the remodeling of intracellular calcium homeostasis during cell differentiation (105).

### Role of IP_3_Rs in the remodeling of calcium signaling

Inositol triphosphate receptors are ER-resident calcium channels that form tetrameric assemblies and thereby release calcium into the cytosol from the ER. IP_3_Rs play an important role in calcium signaling and homeostasis. IP_3_Rs consist of three subtypes (IP_3_R_1_, IP_3_R_2_, and IP_3_R_3_) that are differentially expressed in specific cell types (91, 107, 108). IP_3_, which is generated via the phospholipase C pathway and is an agonist of IP_3_Rs, directly binds to the cytosolic region of all three IP_3_R subtypes and enhances calcium release (109, 110). The activity of IP_3_Rs is also positively or negatively regulated by endogenous factors including calcium, ATP, and pH (111, 112). Interestingly, some reports have demonstrated that kinase and cytosolic proteins can regulate the activity of IP_3_Rs (113–115). It is reported that serine/threonine kinase Akt/protein kinase B phosphorylates IP_3_Rs, resulting in inhibition of calcium release activity of IP_3_Rs (113, 114). In addition, Bcl-2 family protein BAX and BAK positively regulates calcium release from the ER through a decrease of IP_3_Rs phosphorylation (115).

Recent studies have shown alterations in the activity and expression of IP_3_Rs during various processes of tumor cells including survival, growth, proliferation, invasion, and metastasis (116–127). In breast cancer cells, activation of IP_3_Rs, especially IP_3_R_3_, is enhanced by ATP, thereby promoting cell growth through regulation of the spatiotemporal pattern of intracellular calcium (116–119). Moreover, enhanced expression of IP_3_R_1_ and IP_3_R_3_ is also involved in the epithelial-mesenchymal transition (EMT) of breast cancer (117, 120). In addition to breast cancer, IP_3_R_3_ is specifically overexpressed in gastric cancer cells (121). Inhibition of IP_3_R_3_ by an antagonist of IP_3_Rs attenuates their proliferation and induces apoptosis (121). Additionally, although the IP_3_R subtype remains unclear, colorectal cancer (122), lung cancer (123, 124), melanoma (125), and insulinoma (126, 127) also show enhanced IP_3_R activity and expression, leading to the remodeling of calcium signaling. Therefore, IP3Rs might be therapeutic targets for treatment of cancer.

## Lipid Signaling from the ER

**Figure 3:**
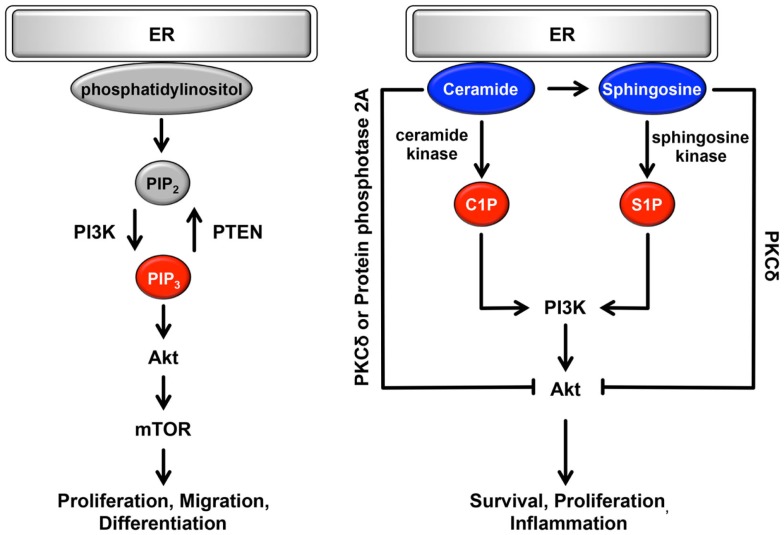
**Role of the ER lipid signaling pathway**. In the ER, glycerophospholipids and phosphatidylinositol are synthesized. Phosphatidylinositol is converted to highly phosphorylated forms. Conversion of PIP_2_ to PIP_3_ is promoted by PI3K, resulting in the regulation of cell proliferation, cell migration, and cell differentiation through the induction of PI3K-Akt-mTOR signaling. Sphingosine is also synthesized on the cytosolic surface of the ER through the *de novo* biosynthesis of ceramide. Both ceramide and sphingosine inhibit Akt activity via the activation of PKCδ and protein phosphatase 2A. Conversely, C1P and S1P antagonize the effects of ceramide and sphingosine and activate Akt, consequently contributing to cell survival, cell proliferation, and inflammation. Pro-oncogenesis-related lipids are indicated in red. Anti-oncogenesis-related lipids are indicated in blue.

The ER can be classically divided into two types, rough ER (rER) and smooth ER (sER), which have different functions and morphology (128). The rER is studded with ribosomes on the membrane surface; it forms a membranous sheet and plays an important role as a major site of protein synthesis. By contrast, the sER is ribosome-free and exists as a tubular or reticular network throughout the cytoplasm (128). A large number of reports have demonstrated that sER is the site of lipid biogenesis. Among various lipids and phospholipids, glycerophospholipids and sphingolipids are essential as major components of biological membranes, and they are also important as signaling molecules (129, 130). These signaling lipids play important roles in cellular processes such as cell proliferation, migration, metabolism, inflammation, and apoptosis (Figure 3). In addition, both rER and sER are composed mainly of glycerophospholipids [i.e., phosphatidylcholine, phosphatidylethanolamine, phosphatidylinositol (PI), and phosphatidyleserine], and sphingolipids [i.e., sphingosine-1-phosphate (S1P) and sphingosine] (130). In these phospholipids, including both saturated and unsaturated fatty acids are usually disturbed in cancer cells (36–38). Recent studies have demonstrated that changing the balance between membrane phospholipid saturation and unsaturation activates UPR signaling (10, 12). Furthermore, alterations in fatty acid unsaturation in membranes are implicated in cancer (131). Based on these findings, it is believed that the UPR-regulated signaling lipids or their precursors may have important roles in tumorigenesis and in the maintenance of cancer cell metabolism.

### Role of lipid signaling via glycerophospholipids

Glycerophospholipids are glycerol-based phospholipids consisting of a polar group and hydrophobic chains. In general, glycerophospholipids are classified by differences in polar group (37, 130, 132). Among the glycerophospholipids, phosphatidylcholine, phosphatidylethanolamine, and PI are synthesized in the ER. Glycerol and fatty acids are transformed into phospholipid precursors, including triglycerides and diacylglycerol phosphate, by ER-resident enzymes. During the next synthesis step, on leaflets of the ER membrane, these phospholipid precursors are dephosphorylated and then converted to phosphatidylcholine or phosphatidylethanolamine. PI is also synthesized from l-myo-inositol and CDP-diacylglycerol through PI synthase in the ER. Synthesized PI can be converted to several highly phosphorylated forms called phosphoinositides. They are localized mainly in the plasma membrane and play important roles in various signaling pathways and vesicle trafficking (49, 130, 132). Among the various types of phosphoinositides, the plasma membrane-resident phosphoinositides PI-4,5-bisphosphate [PI(4,5)P_2_] and PI-3,4,5-bisphosphate [PI(3,4,5)P_3_] are specifically involved in cell growth- and survival-related intracellular signaling (37). In response to stimulation by growth factors, conversion of PIP_2_ to PIP_3_ is promoted through phosphorylation by the class I phosphoinositide 3-kinase (PI3K) (37, 133). This conversion is transient and dramatic, and it enhances the binding of Akt to PI(3,4,5)P_3_, and thereby to phosphorylated Akt, resulting in activation of important cascades, including mammalian target of rapamycin (mTOR) signaling (133, 134) (Figure 3). mTOR signaling is an oncogenic PI3K pathway that regulates protein synthesis, cell proliferation, migration, differentiation, and the cell cycle (134–137). Conversion of PI(3,4,5)P_3_ to PI(4,5)P_2_ occurs through dephosphorylation via the phospholipid phosphatase PTEN and is antagonized by PI3K pathway (138, 139). Thus, cellular functions are maintained through a physiological balance of phosphorylation and dephosphorylation by PI3K and PTEN, respectively. Interestingly, several cancers frequently show constitutively active PI3K mutations or loss-of-function mutations in PTEN, resulting in an increase in PI(3,4,5)P_3_ that in turn contributes to oncogenesis (37, 140, 141). These mutations are particularly observed in ovarian, colon, breast, and gastric cancer (142). Although the amount of additional PI, a precursor of PI(4,5)P_2_ and PI(3,4,5)_3_, remains poorly understood, PI3K, Akt, and mTOR may be therapeutic targets for various cancers.

### Role of lipid signaling via sphingolipids

Sphingolipids are sphingoid-based phospholipids that are produced through the *de novo* biosynthesis of ceramide, which is generated by a condensation reaction involving serine and palmitoyl-CoA via serine palmitoyl transferase (37, 107, 109). This biosynthesis occurs on the cytosolic surface of the ER. Then, ceramide is coordinated by various enzymes, including ceramide kinase, glucosylceramide synthase, galactosyltransferase, sphingomyelin synthase, and ceramidase, consequently producing ceramide 1-phosphate (C1P), glycosphingolipids, sulphatide, sphingomyelin, sphingosine, and S1P. Conversely, ceramide is synthesized from these products via several metabolic pathways (37, 107, 109). Among sphingolipids, ceramide, C1P, sphingosine and S1P are potent signaling molecules and have important roles in cellular processes. Ceramide and sphingosine are involved in anti-oncogenic processes such as apoptosis, cell-cycle arrest, and cellular senescence (37, 143–146) (Figure 3). By contrast, C1P and S1P antagonize the pro-apoptotic effects of ceramide and sphingosine and contribute to survival, proliferation, and inflammation (37, 38, 143–145). Ceramide and sphingosine can attenuate Akt activity (37, 38); they directly activate PKCδ, which inhibits the translocation of Akt to the plasma membrane, leading to suppression of Akt activity. As another inhibitory mechanism, ceramide dephosphorylates Akt through the activation of cytosolic protein phosphatase 2A. By contrast, C1P and S1P are positive regulators of the Akt signaling pathway via activation of PI3K (37, 143, 146). Interestingly, S1P is linked to endothelial growth factor-, platelet-derived growth factor-, and transforming growth factor β-related signaling pathways (146–148). Consequently, Akt, ERK1/2, and PKC-β are activated by crosstalk between S1P and growth factors. Surprisingly, it has been reported that several sphingolipid synthases and metabolites are dysregulated in many types of cancer. Especially in leukemia, melanoma, breast, ovarian, and colon cancers, sphingosine kinase is substantially upregulated, resulting in increased generation of S1P (149–155). Conversely, the production of ceramide is suppressed by increased ceramide kinase in liver and breast cancer (156, 157). Furthermore, in the *de novo* biogenesis of ceramide, the expression of serine palmitoyl transferase, which is localized on the surface of the ER, is decreased (149). Based on these findings, it is possible that cancers evade the anti-oncogenic effects of sphingolipids via the dysregulation of sphingolipid metabolism including the upregulation of anti-apoptotic sphingolipids.

## Signaling Pathways from the MAM

As noted above, in addition to the rER and sER, recent reports have defined a subdomain of the ER that is a site of direct communication between the ER and mitochondria, called the MAM (49). It is believed that this tether is important for lipid and calcium trafficking between the ER and mitochondria to regulate lipid metabolism and mitochondrial calcium homeostasis (25–30, 49). Several proteins involved in lipid synthesis and trafficking are enriched at the MAM. Acyl-CoA synthase 4, acyl-CoA cholesterol acyl transferase 1, phosphatidyl serine synthase, phosphatidylethanolamine *N*-methyltransferase 2, and acyl-CoA:diacylglycerol acyltransferase 2 have been identified as MAM-enriched enzymes (25, 32–35). Among these enzymes, acyl-CoA synthase 4 is well known as a marker protein of the MAM. Furthermore, it has also been reported that the MAM is important for the *de novo* biogenesis of phospholipids and for their transport between the ER and mitochondria (25, 32–35). At the MAM, IP_3_R binds to the outer-mitochondrial-membrane-localized calcium channel VDAC1 via the cytosolic chaperone GRP75, leading to the transfer of calcium to the mitochondrion (22). Furthermore, the ER chaperone sigma-1 receptor is also localized in the MAM and can interact with IP_3_R (158). This interaction is related to the stability of IP_3_R and sustains the calcium transfer from the ER to the mitochondria. SERCA2b is also localized at the MAM and regulates the import of calcium into the ER (159). More recently, in addition to proteins involved in lipid and calcium trafficking, a variety of MAM-resident proteins, including proteins related to oncogenic signaling, have been characterized (Figure 2; Table 1).

**Table 1:** **MAM-resident proteins associated with a variety of signaling pathways and cancers**.

Functional role	Key MAM proteins or complexes	Specific effect	Expectation of pro- or anti-oncogenesis	Reference
Apoptotic signaling	PML	Involvement with calcium homeostasis and mitochondrial calcium overload	Anti	(167)
	S1T	Induction of mitochondrial calcium overload-induced apoptosis	Anti	(170)
	PTEN	Enhancement of ER-mitochondria calcium transfer though regulation of IP_3_R	Anti	(171)
Inflammation signaling	NLRP3	NLRP3 inflammasome forms at the MAM upon NLRP3 activation	Anti	(174)
Antiviral innate immune response	MAVS-RIG-I	The MAVS-RIG-I signaling induces from the MAM during virus infection	Anti	(183)
Autophagic signaling	Atg14L	Essential for the origin of autophagosome membrane	Anti	(190)
Cellular metabolism-related signaling	mTORC2	MAM-resident mTORC2 controls the MAM integrity and mitochondrial functions	Pro	(194)
UPR signaling	PERK	PERK plays a role of in ER-mitochondria tethering	Pro	(186)
	MFN2	MFN2 can be interacts with PERK and regulates PERK-mediated UPR	Pro	(187)

## Apoptotic Signaling Pathway at the MAM

Mitochondria are intracellular power plants that produce ATP and many biosynthetic intermediates while also contributing to the metabolism of amino acids and lipids and to the maintenance of intracellular calcium homeostasis (160–164). Mitochondria also have non-canonical functions such as the induction and amplification of apoptotic cell death (165, 166). Interestingly, recent reports have shown that the MAM functions in the apoptotic signaling pathway through dysregulation of calcium transfer from the ER to mitochondria. Mitochondrial calcium uptake is enhanced during apoptosis. Promyelocytic leukemia (PML) protein, which is involved in calcium homeostasis and apoptosis, is localized at the MAM and functions there (167). PML normally forms complexes with IP_3_Rs, Akt, and protein phosphatase 2, resulting in a reduction in the hyper-phosphorylation of IP_3_R_3_, which in turn results in mitochondrial calcium overload through an increase in calcium release from the ER (167). Interestingly, PML is often decreased or dysregulated in cancer cells, leading to an escape from mitochondrial calcium-mediated apoptosis (168, 169). Thus, it is considered that MAM-resident PML is involved in anti-oncogenesis and is a tumor suppressor.

Another MAM-resident protein involved in apoptosis, the truncated form of the SERCA (S1T), has been shown to induce pro-apoptotic mitochondrial calcium overload (170). High expression of S1T increases basal mitochondrial calcium and triggers abnormal mitochondrial structure and function, resulting in apoptotic cell death. S1T knockdown results in an impairment of mitochondrial calcium overload-induced apoptosis. Moreover, S1T has been shown to be involved in ER stress (170). Taken together, it is possible that S1T is important for cell fate through ER-mitochondrion calcium transfer-induced ER stress.

PTEN dephosphorylates PI(3,4,5)P_3_, converting it to PI(4,5)P_2_, thereby attenuating the oncogenic PI3K signaling cascade (138, 139). Thus, PTEN has been considered a tumor suppressor protein. Interestingly, some PTEN proteins are localized at the MAM and function in calcium transfer from the ER to mitochondria and in pro-apoptotic mitochondrial calcium overload (171). PTEN knockdown impairs calcium release from the ER, decreases the cytosolic and mitochondrial calcium levels, and thereby reduces cytochrome C release and caspase 3 cleavage. These results indicate that ablation of PTEN attenuates calcium-dependent apoptotic cell death. By contrast, overexpression of a specific chimeric PTEN that is localized at the cytoplasmic surface of the ER membrane enhances ER-mitochondrion calcium transfer and the vulnerability of cells to arachidonic acid-induced apoptosis. This effect of PTEN is dependent on IP_3_R function. PTEN interacts with IP_3_Rs, thereby inhibiting their hyper-phosphorylation and consequently increasing calcium release from the ER. Surprisingly, the protein phosphatase activity of MAM-resident PTEN is independent of its lipid phosphatase activity (171). Based on these findings, PTEN is thought to prevent oncogenic signaling through its canonical and non-canonical activities.

## Inflammatory Signaling Pathway at the MAM

Inflammasomes are important large multiprotein complexes of the innate immune system that act as molecular platforms for immune defenses against microbial-, viral infection- and stress-mediated cellular danger signals via maturation or release of pro-inflammatory cytokines including IL-1β and IL-18 (172, 173). The components and mechanism of activation of the NOD-like receptor family pyrin domain-containing 3 (NLRP3) inflammasome have been particularly well analyzed (172, 173). In response to microbial stress, NLRP3 becomes oligomerized prior to its activation. It interacts with apoptosis-associated speck-like protein containing a CARD (ASC) and recruits procaspase-1, which in turn is converted to active caspase-1, leading to the generation of mature IL-1β (172, 173). NLRP3 protein has been reported to be enriched both in the ER and at the MAM (174). In addition, NLRP3 is co-localized with ASC proteins at the MAM when NLRP3 is activated by stimulation with nigericin or monosodium urate, which are known as inflammasome inducers. Thus, the NLRP3 inflammasome may be formed at the MAM. The detailed mechanisms, such as how and why the NLRP3 inflammasome is localized at the MAM, remain unclear. However, it has been reported that ROS generation from damaged mitochondria is required for NLRP3 inflammasome activation (174). There is a possibility that NLRP3 at the MAM senses mitochondrial function. The role of inflammasomes includes not only immune responses but also cancer pathogenesis. In various cancers, the NLRP3 inflammasome has been shown to be involved in the prevention of tumorigenesis, malignancy, and growth (175). *NLRP3^−/−^* mice exhibit tumors of increased mass, number, and size. The deletion of the NLRP3 inflammasome components ASC and caspase-1 produces a similar phenotype. These findings indicate that the NLRP3 inflammasome has anti-tumor functions (175). Furthermore, it has been reported that NLRP3 inflammasome components, including NLRP3, ASC, and procaspase-1, are downregulated in hepatocellular carcinoma (HCC) (176). This downregulation is dependent on a decrease in their mRNA levels. Interestingly, the expression pattern of NLRP3 inflammasome components changes dynamically in the development stage of hepatocarcinogenesis. Although the components of the NLRP3 inflammasome components are expressed at low levels in the healthy liver, they are upregulated in hepatitis and hepatic cirrhosis. However, their expression levels substantially decrease during hepatocarcinoma development. From these findings, this report concluded that dynamic regulation of the expression of NLRP3 inflammasome components is required for HCC development and progression. The MAM may be a central platform for NLRP3 inflammasome-mediated anti-tumorigenic functions.

## Antiviral Innate Immune Response at the MAM

Retinoic acid-inducible gene I (RIG-I) is a cytosolic pathogen recognition receptor that contributes to the innate immune response against viral infection via its adaptor protein mitochondrial antiviral signaling (MAVS) (177, 178). RIG-I-MAVS signaling triggers the production of type I IFN and pro-inflammatory cytokines and results in interference with viral replication (177, 178). Viral RNA genome fragments trigger apoptotic cell death in cancer cells through the pro-apoptotic protein, TNF-related apoptosis-including ligand and NOXA, which are induced by RIG-I-MAVS signaling (179). The RIG-MAVS signaling pathway is believed to function in the cellular antitumor immune response (180). Some groups have reported that MAVS can be localized to the mitochondria or peroxisomes (181, 182). The main localization site of MAVS has been shown to be the MAM (183). Moreover, RIG-1 is recruited to the MAM-resident MAVS upon virus infection, leading to the activation of RIG-I-MAVS signaling (183). When the formation of the MAM is inhibited by MFN2 knockdown, MAVS localization increases at the peroxisomes, resulting in the enhancement of IFN-β promoter activity via RIG-I-MAVS signaling. These results suggest that the MAM can regulate the localization and signaling of MAVS. Surprisingly, when cells are infected with hepatitis C virus, NS3/4A protease selectively cleaves the MAM-resident MAVS to avoid the antiviral response. Because cleaved MAVS does not have antiviral activity, it suppresses IFN-β promoter activity. The MAM may function as a platform for innate immune signaling.

## Role of UPR Proteins at the MAM

Several UPR-related proteins, including ER chaperones, calcium channels, and ER-resident oxidoreductase, have been shown to reside at the MAM (159, 184, 185). MAM-resident PERK has been shown to possess heterogeneous roles. For example, PERK is important for the maintenance of the MAM and triggers ROS-mediated mitochondrial apoptosis (186). PERK deficiency leads to ER fragmentation and aberrant calcium release. This functional change of the ER requires defective MAM formation due to PERK deletion. Interestingly, transient transfection with a kinase-dead PERK mutant can reconstitute the formation of the MAM, indicating that the role of PERK in MAM formation is independent of its kinase activity. Furthermore, this PERK function is involved in mitochondrial sensitization during ROS-mediated stress and induces ROS-mediated apoptosis. PERK has been shown to interact directly with MFN2, which forms a molecular bridge between the ER and mitochondria (187). MFN2 deletion induces ER stress and activates the three branches of UPR signaling; the PERK, ATF6, and IRE1α pathways (187). Although UPR signaling mediates the pro-apoptotic pathway under prolonged ER stress conditions, MFN2 deletion results in the inhibition of ER stress-induced apoptosis. This MFN2-mediated apoptosis requires PERK. Furthermore, when PERK is deleted in MFN2-deleted cells, ROS production and mitochondrial calcium overload are reduced, and mitochondrial morphology is improved. Therefore, in addition to its role in the UPR, PERK is involved in the regulation of mitochondrial morphology and function. Based on these reports, it is believed that PERK has multiple functions through its canonical and non-canonical activities both in the ER and at the MAM. Although the role of MAM-resident PERK in oncogenesis remains unclear, it is known that PERK is involved in the adaptation of cancer cells to tumor microenvironmental challenges. Thus, it is possible that MAM-resident PERK also has a pathological function and might be a therapeutic target in cancer.

## Role of the MAM in the Autophagic Signaling Pathway

Autophagy is one of the proteolytic systems that contribute to the lysosome-mediated degradation pathway required for maintaining intracellular homeostasis (188). Defects in autophagy result in enhanced tumorigenesis, suggesting that this system plays an important role in tumor suppression (189). Autophagy is tightly regulated by a set of autophagy-related proteins (Atgs) that mediate the formation of autophagosomes (188). Autophagy requires two ubiquitin-like conjugation systems mediated by the covalent binding of Atg12 to Atg5 and a linkage between LC3 and phosphatidylethanolamine. The Atg12–Atg5 conjugate interacts with Atg16 and organizes a non-covalent multimeric complex. It localizes in nascent autophagosomes and functions as an E3 ligase of LC3, resulting in autophagosome formation. More recently, the MAM has been discovered to be the origin of autophagosome formation (190). Among the many Atg proteins, Atg14L, which is well known as a pre-autophagosome marker, re-localizes to the MAM during starvation, and Atg5 then localizes to the same site during autophagosome formation. Furthermore, in terms of the mechanism of autophagosome formation at the MAM, it has been reported that MAM-resident Atg14L interacts with the ER-resident SNARE protein syntaxin 17. Although the detailed mechanism of autophagosomal membrane generation is poorly understood, this event may be regulated via the coordination of MAM-resident proteins.

## Cellular Metabolism-Related Signaling Pathway at the MAM

Mammalian target of rapamycin plays an important role in the regulation of various cellular processes (134). It forms two functionally distinct multi-protein complexes, mTOR complex 1 (mTORC1) and mTORC2 (135, 191). mTORC1 is composed of mTOR, Raptor, mLST8, and DEPTOR, and it is sensitive to rapamycin. This complex contributes to the regulation of protein synthesis and cell growth (134, 135, 191, 192). mTORC2 is composed of mTOR, Rictor, Sin1 mLST8, and DEPTOR, and it regulates AGC subfamily kinases such as Akt and consequently controls cell growth, cell proliferation, cell spreading, and organization of the actin cytoskeleton (134, 135, 191, 193). Furthermore, in addition to these roles, mTORC2 contributes to cell survival, cell metabolism, and cell migration in cancer (193). Thus, mTORC2 is considered a key regulator of cancer metabolic reprograming, including glycolytic metabolism, lipid metabolism, glutamine metabolism, and ROS metabolism, for protection against microenvironmental challenges (193). Interestingly, a recent report has shown that mTORC2 is localized to the MAM during growth factor stimulation (194).

Mitochondria-associated ER membrane-resident mTORC2 can interact with the IP_3_R-GRP75-VDAC1 complex, which is a molecular bridge at the MAM. Defective mTORC2 results in a decrease of the ER-mitochondrion contact site, producing mitochondrial dysfunction including abnormal ATP production and mitochondrial calcium overload. Mechanistically, mTORC2 phosphorylates IP_3_Rs, hexokinase 2, and phosphofurin acidic cluster sorting protein 2 through the phosphorylation of Akt. The phosphorylation of these molecules mediates mitochondrial functions and the integrity of the MAM. Furthermore, activation of the mTORC2-Akt-HK2 pathway at the MAM is involved in energy metabolism and cell survival. Thus, in addition to the canonical roles of mTORC2, MAM-resident mTORC2 may control mitochondrial function and cellular metabolism.

## Conclusion

**Figure 4:**
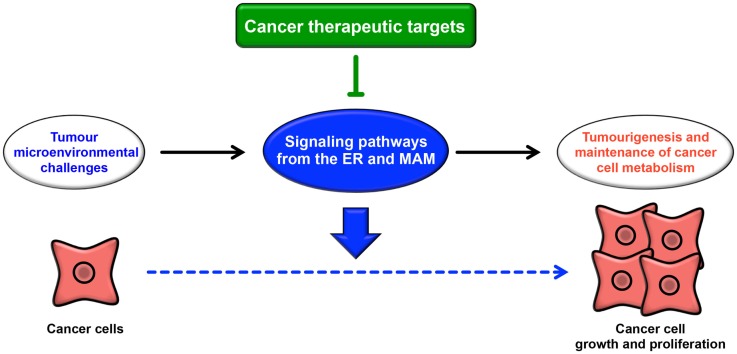
**Roles of signaling pathways from the ER and MAM in cancer**. Cancer cells are constitutively exposed to microenvironmental challenges, resulting in the remodeling of signaling pathways from the ER and MAM. Dysregulation of these signaling pathways directly or indirectly contributes to tumorigenesis and the maintenance of cancer cell metabolism. Thus, these pathways may lead to novel therapeutic strategies for various cancers.

The ER extends throughout the cytoplasm and has heterogeneous morphology and various functions. In addition to newly synthesized protein folding, calcium homeostasis, and phospholipid synthesis, the ER is a platform for various intracellular signaling pathways. The current understanding of these signaling pathways suggests that multiple functions of the ER are relevant to cancer pathogenesis. The signaling pathways from the ER contribute not only to oncogenesis but also to tumor suppression. The UPR is well known to contribute to the adaptation to tumor microenvironmental challenges through its constitutive activation (Figure 4). Furthermore, the maintenance of calcium homeostasis in cancer cells is critical for tumorigenesis and tumor cell survival. Similarly, lipid metabolism is also affected, inducing abnormal glycerophospholipid- and sphingolipid-related signaling, thereby leading to tumor cell survival. More recently, it has been reported that the MAM is a platform for various signaling pathways, including apoptotic signaling, inflammatory signaling, antiviral innate immune responses, autophagic signaling, and metabolism-related signaling pathways. Interestingly, these signaling pathways are closely related to both pro- and anti-oncogenic processes. Although research into the relationship between the MAM and cancer has only recently begun, it is expected that further characterization of the signaling pathways from the ER, especially from the MAM, will reveal new insight into novel cancer therapies (Figure 4).

## Conflict of Interest Statement

The authors declare that the research was conducted in the absence of any commercial or financial relationships that could be construed as a potential conflict of interest.
